# Comparative analysis of rhizobacterial communities across five medicinal plants in Xinjiang

**DOI:** 10.3389/fmicb.2026.1785383

**Published:** 2026-04-22

**Authors:** Jing Wu, Ru Bai, Oren Akhberdi, Liping Xu

**Affiliations:** 1 College of Biological Sciences and Technology, Yili Normal University, Yining, Xinjiang, China; 2 Xinjiang Key Laboratory of Lavender Conservation and Utilization, Yining, Xinjiang, China

**Keywords:** bacterial community, high-throughput sequencing, medicinal plants, rhizosphere, soil physicochemical properties

## Abstract

Five medicinal plants with various therapeutic effects in Xinjiang (*Helichrysum thianschanicum* Regel, *Taraxacum kok-saghyz* Rodin*, Artemisia rupestris* L.*, Arnebia euchroma* Johnst, *and Hyssopus officinalis* L.) are considered as promising raw materials of pharmaceutical, cosmetic, and fragrance industries. Understanding the characteristics of rhizosphere bacterial communities can be critical in regulating the growth process of these medicinal plants. To clarify the plant-specificity of rhizobacterial assemblages and related driving factors of five medicinal plants, we compared their rhizosphere bacterial communities with those of non-planted soil (CK). Soil physicochemical properties were analyzed, and bacterial communities were characterized using high-throughput sequencing of the 16S rRNA gene V3–V4 region. Results showed that rhizosphere soils of medicinal plants had significantly higher organic matter (OM), total nitrogen (TN), and total phosphorus (TP) than CK, with TP increasing by 2.6–2.9 times. Each medicinal plant rhizosphere harbored a highly specific bacterial community, with unique Amplicon Sequence Variants (ASVs) accounting for 34.04–46.70% of total ASVs, while only 449 core ASVs were shared among all five plants. At the phylum level, *Proteobacteria*, *Bacteroidota*, and *Acidobacteriota* dominated the rhizosphere, in contrast to the dominance of *Actinobacteriota* in CK. *Chitinophagaceae*, *Sphingomonas*, *Pseudomonas* and *RB41* were selectively enriched in different plant rhizospheres. Total potassium (TK), TN, and TP were the key edaphic drivers, with TK negatively correlated with bacterial diversity, and TN/TP regulating the distribution of dominant genera. This study demonstrates high plant-specificity of rhizobacterial communities in Xinjiang medicinal plants and the dominant role of soil TK, TN, and TP, providing a scientific basis for the precise cultivation of medicinal plants and the development of specialized microbial agents.

## 1 Introduction

Medicinal plants are valuable resources for pharmaceuticals, cosmetics, and fragrances due to their diverse bioactive compounds (Ali et al., 2023). However, maintaining stable yield and phytochemical quality in cultivated medicinal plants remains a major challenge, limiting their commercial and therapeutic potential (Asaf et al., 2020).

The rhizosphere, as a dynamic interface between plant roots and soils, harbors complex bacterial communities that interact closely with plants. Root exudates including enzymes, organic acids, secondary metabolites and litter can serve as nutrient sources for bacteria, while rhizobacteria enhance plant nutrient uptake, disease resistance, and stress tolerance (Bailey et al., 2013; Berg and Smalla, 2009; Broeckling et al., 2008; Carrión et al., 2019; Chen et al., 2024). For instance, specific correlations have been found between root exudate metabolites (e.g., lignans, organic acids) and dominant rhizosphere bacterial phyla such as *Acidobacteria* and *Actinobacteria* in several medicinal plants (Qu et al., 2024). Moreover, rhizosphere microorganisms dominated by *Proteobacteria* bacterial community and *Ascomycota* fungal community can effectively promote nitrogen and phosphorus metabolism in cultivated *Rheum officinale* (Wang et al., 2025). *Paraburkholderia* in *Coptis chinensis* has been identified as a probiotic flora, which can significantly inhibit the main pathogens of root rot via activating plant immune responses (Cao et al., 2025). *Bacillus flexus* KLBMP 4941 bacterium can facilitate the growth of *Limonium sinense* under salt stress (Xiong et al., 2020). These results underscore the critical role of rhizosphere microbiota in the growth and quality of medicinal plants.

Plants can selectively shape their rhizosphere bacterial communities through species-specific root exudation patterns (Bulgarelli et al., 2012; Trivedi et al., 2020; Fitzpatrick et al., 2018; Lundberg et al., 2012; Wippel et al., 2021; Edwards et al., 2015). For example, significantly distinct rhizobacterial community structures were observed in different plant species under the identical planting region, owing to varied root exudates (De Vries et al., 2020; Doornbos et al., 2012). In addition, soil physicochemical properties can further affect the composition and function of rhizosphere microbiomes, such as nutrient availability and pH (Jansson and Hofmockel, 2020; Philippot et al., 2024; Delgado-Baquerizo et al., 2020; Hu et al., 2024). Particularly, soil nitrogen (N), phosphorus (P), and potassium (K) have been identified as key drivers of microbial diversity and community structure in plant rhizosphere (Chen et al., 2006; Fageria and Stone, 2006). Therefore, it is essential to construct the relationship of plant species, soil factors and rhizosphere bacterial communities for targeted agricultural management.

Xinjiang’s unique ecosystems in northwestern China provide ideal habitats for multiple valuable medicinal plant species. Among them, *Helichrysum thianschanicum Regel* (HTR), *Taraxacum kok-saghyz* Rodin (TKR), *Artemisia rupestris* L. (ARL), *Arnebia euchrom*a Johnst (AEJ), and *Hyssopus officinalis* L. (HOL) are particularly notable for their pharmacological activities, including anti-inflammatory, antioxidant, antimicrobial, and anticancer properties (Ho et al., 2017; Huang and Lu, 2022; Sharopov et al., 2018; Tahir et al., 2018). Despite their economic and medicinal importance, the rhizosphere bacterial communities of these medicinal plants remain poorly understood. Moreover, it is unclear whether they recruit plant-specific bacterial assemblages and how soil nutrient factors govern microbial characteristics.

We hypothesized that (1) distinct medicinal plants enrich plant-specific bacterial taxa, and (2) soil nutrient availability, particularly K, N, and P, are primary drivers shaping these communities. The study aimed to: (i) compare the rhizosphere soil physicochemical properties among the five medicinal plants and non-planted soil; (ii) characterize the diversity and composition of rhizosphere bacterial communities using high-throughput sequencing of the 16S rRNA gene; and (iii) identify key edaphic factors and selectively enriched bacterial taxa associated with each plant species. The results could provide a reference for scientifical cultivation of the premium and high-yield medicinal plants.

## 2 Materials and methods

### 2.1 Study site

The study site is located in an experimental planting area (approximately 70 acres; 43°09′N, 80°08′E) located within the Agricultural Science and Technology Demonstration Zone in Zhaosu County, Xinjiang Uygur Autonomous Region, China. To minimize the influence of geographic variability, all samples were collected from adjacent regions within above experimental field. The zone hosts more than 80 varieties of economic and medicinal plants. The experimental site had previously been maintained as monocropped farmland, thus providing a uniform baseline in soil type, physicochemical properties, fertility, and moisture prior to the cultivation of medicinal plants. This homogeneity helped eliminate potential confounding effects from inherent soil heterogeneity during the study.

Zhaosu County is situated in the southwest of the Ili Kazakh Autonomous Prefecture. Due to its high altitude (around 1800 m), the county experiences a predominantly cold climate ranging from semi-arid to semi-humid within the continental temperate mountainous zone. The region records an average annual temperature between −1 °C and 10 °C, receives about 2,699 h of sunshine annually, and has an average annual precipitation of 511.8 mm against evaporation of 1261.6 mm. The frost-free period lasts approximately 98 days (Haichar et al., 2008). Additionally, loose chernozems with slight alkalinity are the dominant soil type in this area.

### 2.2 Researched plants

We focused on five perennial medicinal plant species with notable pharmacological or economic value, as detailed below: *Helichrysum thianschanicum* Regel belongs to the family *Asteraceae*. It is distributed in western and northwestern Xinjiang, and exhibits diverse pharmacological activities including heat-clearing, detoxification, antibacterial, anti-inflammatory, antioxidant, anticancer, and antiviral effects (Ho et al., 2017). *Taraxacum kok-saghyz* Rodin, a rosette-shaped perennial herb in the family Asteraceae, is characterized by roots rich in high-quality natural rubber, and also possesses heat-clearing, detoxifying, detumescent, and knot-dissipating properties (Huang and Lu, 2022). *Artemisia rupestris* L. (family *Asteraceae*) is a perennial plant primarily distributed in the Tianshan, Altai, and Kunlun Mountains of Xinjiang, China, with documented anti-inflammatory, antioxidant, antiviral, and antibacterial activities (Kanokratana et al., 2011). *Arnebia euchroma* Johnst is a perennial herbaceous species belonging to the family *Boraginaceae*, found in Xinjiang, western Tibet (China), Pakistan, India, and other Central Asian regions. Its roots have medicinal applications including wound-healing, anti-inflammatory, antibacterial, antitumor, antidiabetic, and antiviral benefits, and it produces naphthoquinone pigments used in the food and cosmetics industries (Lei et al., 2019). Finally, *Hyssopus officinalis* L. (family *Lamiaceae*) exhibits anti-inflammatory properties and is commonly employed to treat conditions such as fever, coughs, colds, and asthma (Li et al., 2018).

### 2.3 Soil sample collection

Rhizosphere soil samples from the medicinal plants were collected using a multipoint mixed sampling method (*n* = 3), with intervals based on plot size. To ensure data comparability, all soil sampling was conducted within May. Five medicinal plants exhibited distinct growth stages during sampling: vegetative stage for HTR, HOL and ARL, flowering stage for TKR and rosette stage for AEJ. Each plant was carefully pulled out and shaken off the loose soil on the roots in a sterile sealed bag, and the soil firmly adhered to the root surface was gently brushed with a brush and then mixed well as a soil DNA extraction sample. Soil as close as possible to the rhizosphere was collected as a physicochemical property test sample. Soil from areas without plant growth served as the control (hereafter referred to as CK), with the top 5 cm of soil and impurities removed before sampling. This sampling process was performed in triplicate to ensure accuracy. The soil samples were transported to the laboratory in an ice box. Following sieving (2-mm mesh) to remove organic debris, stones, and root particles, they were stored at 4 °C and processed within 24 h (Li et al., 2020).

### 2.4 Soil physicochemical properties analysis

Soil physicochemical properties were analyzed in accordance with relevant established Chinese national and agricultural industry standard methods. Soil organic matter (OM) was quantified via the potassium dichromate oxidation method (GB 9834–1988). Total nitrogen (TN) was determined by the semi-micro Kjeldahl method (GB 7173–1987), and total phosphorus (TP) by molybdenum-antimony spectrophotometry (GB/T 9837–1988). Total potassium (TK) was measured using flame photometry following alkali solubilization (GB 9836–1988), while available potassium (AK) was extracted with ammonium acetate and analyzed by flame photometry (NY/T 889–2004). Available phosphorus (AP) was extracted with sodium bicarbonate and determined by molybdenum-antimony colorimetry (NY/T 1121.7–2014). Alkaline hydrolyzable nitrogen (AHN) was analyzed using the alkaline hydrolysis diffusion method (DB51/T 1875–2014). Soil pH was measured potentiometrically in a water suspension (NY/T 1121.2–2006).

### 2.5 Molecular—taxonomy determination

Soil DNA was extracted from each sample using the DNeasy PowerSoil Kit (Qiagen, Hilden, Germany) following the manufacturer’s Quick-Start Protocol. The concentration and purity of the extracted DNA were assessed by 1.0% agarose gel electrophoresis, and the DNA was then diluted with sterile water to a working concentration of 1 ng/μL. The hypervariable V3–V4 region of the bacterial 16S rRNA gene was amplified via PCR with the diluted DNA as the template, using the primer pair 341F (5′-CCTAYGGGRBGCASCAG-3′) and 806R (5′-GGACTACNNGGGTATCTAAT-3′). The resulting amplicons were subsequently subjected to Illumina high-throughput sequencing by Beijing Novogene Biotech Co. Ltd. (Beijing, China).

### 2.6 Data processing and statistical analysis

Raw reads were processed with FLASH (v1.2.11) to merge paired-end reads and remove barcode and primer sequences, yielding Raw Tags. Sequencing artifacts and low-quality sequences were then filtered using Cutadapt (v3.3) and fastp (v0.23.1) to produce high-quality Clean Tags. Chimeric sequences were identified and removed with Vsearch (v2.16.0), resulting in Effective Tags. Amplicon Sequence Variants (ASVs) were inferred, and a feature table was constructed using the DADA2 module in QIIME2 (v2020.6). Taxonomic assignment of ASVs was performed by alignment against the SILVA 138.1 database. For downstream analyses, multiple sequence alignment and data normalization were conducted in QIIME2 to support phylogenetic reconstruction and calculation of alpha diversity indices (Chao1, Shannon, Simpson, and Pielou’s evenness). Rarefaction curves were generated using R (v4.0.3). Additionally, relative-abundance histograms and petal diagrams showing the top 10 taxa at the phylum, family, and genus levels were visualized with the SVG toolkit in Perl (v5.26.2). To explore the functional potential of rhizosphere bacterial communities, KEGG orthologs (KOs) and associated metabolic pathways were predicted using PICRUSt2 (Douglas et al., 2020). Owing to the significant variation in soil OM and TK among plant species (Table 1), two functional categories associated with carbohydrate metabolism-related pathways genes and genes responsive to high potassium stress were selected for further analysis.

**Table 1:** Soil physicochemical properties of five medicinal plants.

Sampled rhizosphere	Soil properties							
	pH	OM (g/kg)	AK (mg/kg)	AHN (mg/kg)	AP (g/kg)	TN (g/kg)	TP (g/kg)	TK (g/kg)
CK	7.50 ± 0.16^ab^	34.74 ± 2.10^b^	479.67 ± 26.58^ab^	127.67 ± 6.66^c^	0.76 ± 0.04^a^	3.37 ± 0.27^b^	0.34 ± 0.27^b^	3.98 ± 0.39^b^
HTR	7.52 ± 0.19^ab^	146.21 ± 27.36^a^	338.33 ± 62.01^b^	148.23 ± 13.49^ab^	0.62 ± 0.21^a^	4.41 ± 0.55^ab^	0.96 ± 0.10^a^	3.31 ± 0.33^c^
TKR	7.30 + 0.11^b^	68.61 ± 16.78^ab^	618.33 ± 87.54^a^	136.43 ± 8.14b^c^	0.75 ± 0.18^a^	4.27 ± 0.29^ab^	1.01 ± 0.05^a^	3.93 ± 0.04^b^
ARL	7.53 ± 0.30^ab^	80.46 ± 4.86^ab^	629.00 ± 65.18^a^	149.67 ± 3.16^ab^	0.67 ± 0.13^a^	4.50 ± 0.07^a^	1.00 ± 0.02^a^	4.38 ± 0.19^ab^
AEJ	7.78 ± 0.09^a^	67.65 ± 8.98^ab^	385.01 ± 57.47^b^	157.57 ± 7.36^a^	0.69 ± 0.10^a^	4.67 ± 0.22^a^	0.90 ± 0.04^a^	4.46 ± 0.12^a^
HOL	7.83 ± 0.02a	61.36 ± 2.26ab	447.00 ± 33.45ab	139.70 ± 10.65bc	0.71 ± 0.02a	4.50 ± 1.03a	1.01 ± 0.01a	3.91 ± 0.15b

Distinct letters (a, b and c) denote significant differences of parameters (*P* < 0.05). This annotation remains consistent in all subsequent data presentations.

## 3 Results

### 3.1 Soil physicochemical properties

The effects of soil environmental factors on rhizosphere bacterial communities were evaluated via the determination of pH, organic matter (OM), total nitrogen (TN), total phosphorus (TP), total potassium (TK), available nitrogen (AN), available phosphorus (AP), available potassium (AK), and alkaline hydrolyzable nitrogen (AHN) (Table 1).

All tested soils were slightly alkaline, with samples from AEJ and HOL exhibiting relatively higher pH values (7.78 and 7.83, respectively) compared with the other samples. Rhizosphere soils exhibited higher concentrations of OM, TN, AHN, and TP relative to the non-planted soil (CK). Notably, TP content in rhizosphere samples showed a significant increase (*p* < 0.05), ranging from 0.90 to 1.01 g/kg compared with 0.34 g/kg in CK, which is presumably attributed to root exudates and the phosphate-solubilizing activities of rhizosphere microorganisms.

Inter-specific differences in soil physicochemical properties were also observed among the medicinal plants. HTR exhibited significantly higher OM content than the other medicinal plant species (*p* < 0.05). Soils associated with ARL and AEJ had high TK content, whereas TKR and ARL soils sustained high AK levels. AHN content in AEJ rhizosphere soil was significantly higher than that in TKR (*p* < 0.05). These results demonstrate that plant growth can effectively modulate soil physicochemical properties.

### 3.2 Soil bacterial diversity in the rhizosphere of five medicinal plants

The sequencing coverage for all samples, as indicated by Good’s coverage index, exceeded 99.77% (Supplementary Table 1). This high coverage corresponds to the plateauing rarefaction curves shown in Supplementary Figure 1, suggesting the sequencing depth was sufficient to reliably characterize the soil bacterial communities. Based on the raw and effective sequence counts, the proportion of high-quality bacterial sequences (effective tags) ranged from 72.28 to 79.47%. The number of Amplicon Sequence Variants (ASVs) per sample, which directly reflects bacterial richness, ranged from 2082 ± 314 to 2,378 ± 441 (Supplementary Table 1). Collectively, these results confirm that the sequencing data provides a robust basis for the analysis of bacterial diversity in the studied samples.

As shown in Supplementary Table 2, no significant differences (*p* > 0.05) were detected in bacterial richness (Chao1) among the groups. Although Shannon index values also showed no statistically significant inter-group variation, a notable numerical trend was observed, with the AEJ sample exhibiting the lowest mean value. In contrast, community evenness (Pielou’s index) varied significantly: HTR had the highest evenness, which was significantly greater than that of AEJ (*p* < 0.05), indicating a more uniform distribution of bacterial taxa. Conversely, AEJ showed significantly lower Pielou’s evenness and Simpson index values compared to several other groups. Together with its numerically lowest Shannon index, these results suggest that the AEJ rhizosphere harbors a bacterial community with reduced diversity, evenness, and ecological stability relative to the other samples.

**Figure 1:**
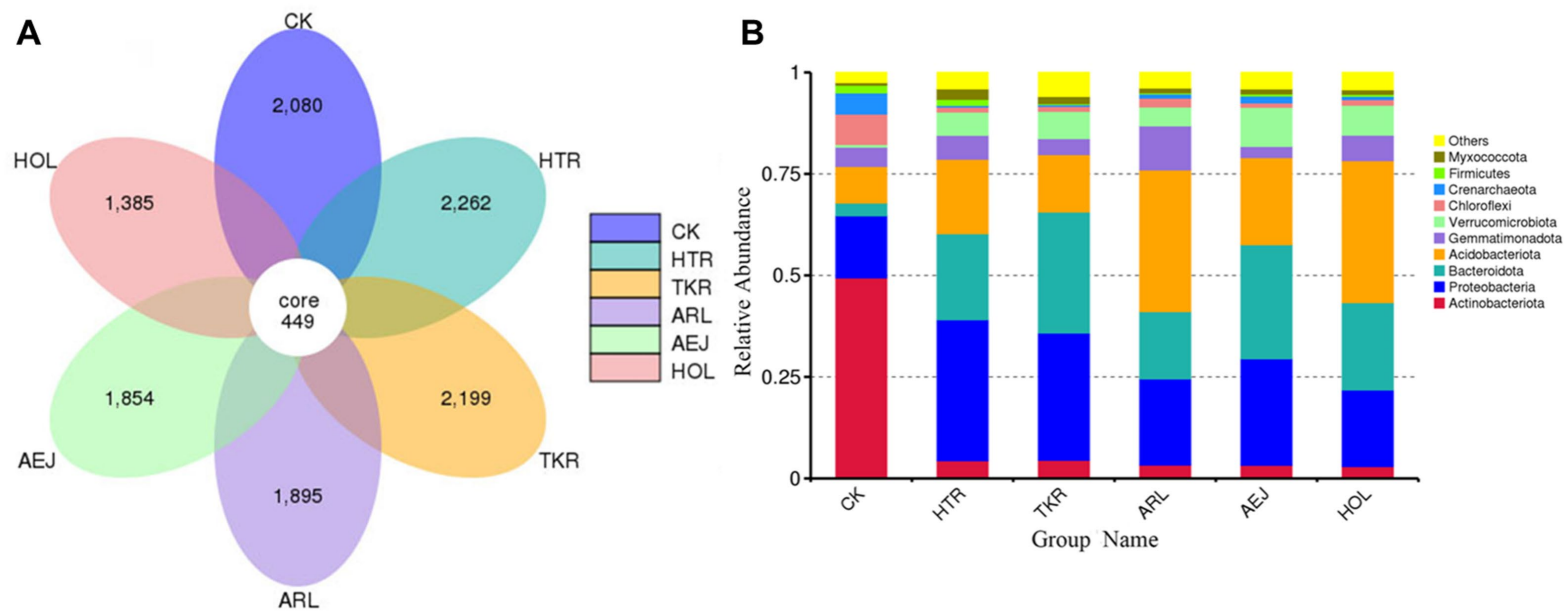
**(A)** Venn diagram showing the unique and shared ASVs (at the 3% evolutionary distance) under five medicinal plants. **(B)** The relative abundance of bacterial taxa at phylum level. Top 10 phyla are shown, and the rest are merged into others.

The shared and unique bacterial Amplicon Sequence Variants (ASVs) across samples were analyzed using a Venn (petal) diagram (Figure 1A). The rhizosphere soil of HTR contained the largest number of unique ASVs (2,262), accounting for 46.70% of its total ASVs, followed by TKR (2,199; 42.95%), ARL (1,895; 41.99%), AEJ (1,854; 40.63%), and HOL (1,385; 34.04%). A core set of 449 ASVs was shared among all five medicinal plant rhizosphere samples, representing 9.27 to 11.03% of the total ASVs in each respective group. In contrast, the non-rhizosphere control soil (CK) harbored a higher proportion of unique ASVs (50.1%) and a comparable proportion of shared ASVs (10.8%). The consistently lower proportion of unique ASVs in the medicinal plant rhizospheres compared to the control soil suggests that plant growth exerts a selective influence, shaping distinct bacterial communities. Furthermore, the significant abundance of unique ASVs in each medicinal plant rhizosphere (*p* < 0.05) confirms the high specificity of their associated bacterial assemblages.

### 3.3 Comparative composition of rhizosphere bacterial communities in five medicinal plants

Analysis of the rhizosphere bacterial community composition at the phylum level revealed distinct profiles compared to non-rhizosphere control (CK) (Figure 1B). In CK, *Actinobacteriota* was the dominant phylum, representing nearly half of the bacterial community. In contrast, the rhizosphere soils of the five medicinal plants were collectively characterized by significantly higher relative abundances of *Proteobacteria* (18.9–34.7%), *Bacteroidota* (16.6–29.8%), and *Acidobacteriota* (14.1–34.9%). Other notable phyla included *Gemmatimonadota*, *Verrucomicrobiota*, *Crenarchaeota*, *Firmicutes*, *Myxococcota*, and *Chloroflexi*. Compared to CK, the medicinal plant rhizospheres exhibited elevated abundances of *Proteobacteria*, *Bacteroidota*, *Acidobacteriota*, *Verrucomicrobiota*, and *Myxococcota*, but reduced proportions of *Actinobacteriota*, *Chloroflexi*, and *Crenarchaeota*.

Substantial variations were also observed among the different medicinal plant rhizospheres. The community in HTR was dominated by *Proteobacteria* and exhibited a notably higher relative abundance of *Firmicutes* and *Myxococcota* compared to the other plant samples. TKR showed the highest relative abundance of *Bacteroidota*. In ARL, *Acidobacteriota*, *Gemmatimonadota*, and *Chloroflexi* were predominant, whereas *Bacteroidota* and *Verrucomicrobiota* were present at lower levels relative to most other rhizospheres. The AEJ sample was characterized by a higher proportion of *Verrucomicrobiota* and *Crenarchaeota* than observed in the other medicinal plants. Furthermore, *Acidobacteriota* maintained a high relative abundance in both ARL and HOL. These results indicate that each medicinal plant harbors a distinct core bacterial assemblage at the phylum level.

**Figure 2:**
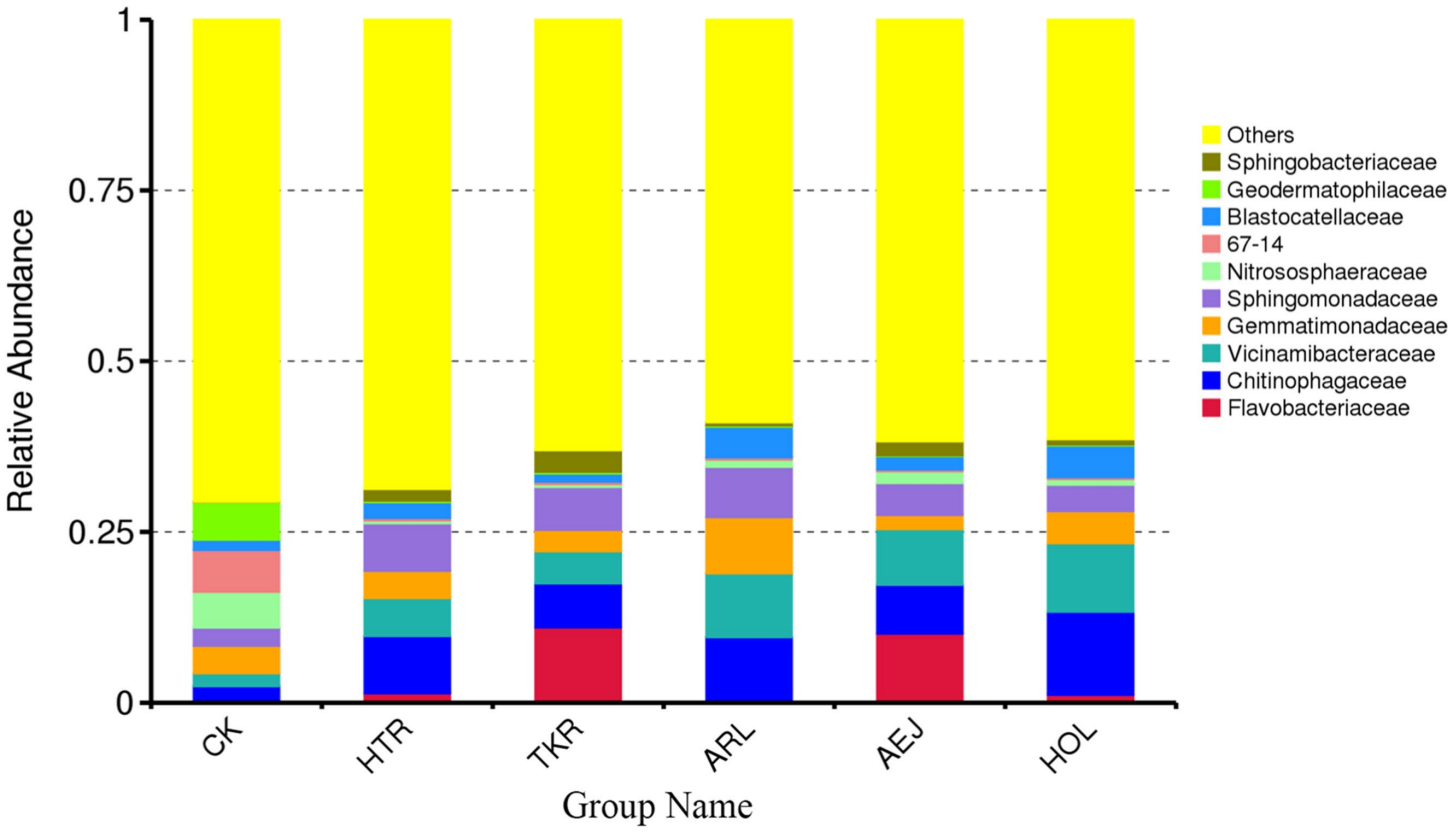
The relative abundance of bacterial taxa at family level. Top 10 phyla are shown, and the rest are merged into others.

At the family level, *Chitinophagaceae* was consistently present among the dominant flora in all five rhizospheres, with its relative abundance being significantly higher than that in the CK (Figure 2). Several families were enriched in the plant rhizospheres: the relative abundances of *Chitinophagaceae*, *Vicinamibacteraceae*, and *Sphingomonadaceae* each exceeded 3.5% across medicinal plant samples but remained below 2.68% in CK. Conversely, families including 67–14, *Geodermatophilaceae*, and *Nitrososphaeraceae* showed lower abundances (mostly <2%) in the rhizosphere soils compared to CK (5.22–6.13%).

Significant compositional differences were also observed among the medicinal plants. *Flavobacteriaceae* was highly abundant only in TKR and AEJ. ARL was characterized by a higher proportion of *Vicinamibacteraceae* and *Gemmatimonadaceae*, while *Blastocatellaceae* was relatively enriched in HOL and ARL. *Sphingobacteriaceae* accounted for the largest share in TKR, and *Microscillaceae* was most prominent in HTR. Collectively, these results indicate that the rhizosphere of each medicinal plant selects a distinct bacterial assemblage at the family level, with *Chitinophagaceae*, *Vicinamibacteraceae*, *Sphingomonadaceae*, and *Flavobacteriaceae* serving as key differential taxa.

**Figure 3:**
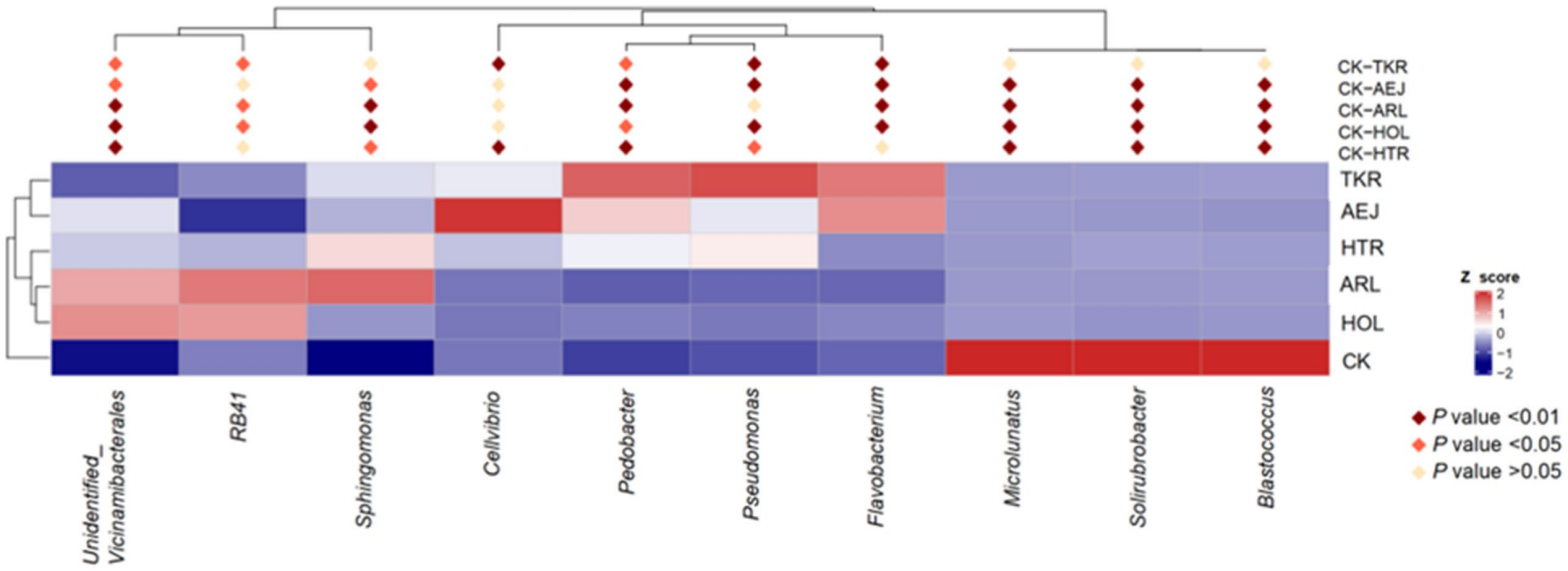
Heatmap depicting the relative abundance of the 10 most differentially dominant genera.

At the genus level, the composition of bacterial communities exhibited distinct patterns between the CK and the medicinal plant rhizospheres (Figure 3). Genera such as *Solirubrobacter*, *Microlunatus*, and *Blastococcus* were relatively more abundant in CK. In contrast, several genera, including unidentified *Vicinamibacterales*, *RB41*, *Sphingomonas*, *Cellvibrio*, *Pedobacter*, *Pseudomonas*, and *Flavobacterium*, were significantly enriched in the rhizospheres of the medicinal plants.

Notable plant-specific enrichment patterns were observed. *Unidentified Vicinamibacterales* and *RB41* were particularly abundant in the ARL and HOL samples. The genus *Sphingomonas* was significantly enriched in the ARL and HTR rhizospheres. *Flavobacterium* and *Pedobacter* were predominantly detected in TKR and AEJ. Furthermore, *Cellvibrio* exhibited a higher relative abundance in ARL compared to other samples, and *Pseudomonas* was a major component of the bacterial community in TKR soil. These results demonstrate that the rhizosphere of each medicinal plant selectively enriches a distinct set of bacterial genera.

### 3.4 Association of key soil physicochemical parameters with microbial diversity

**Figure 4:**
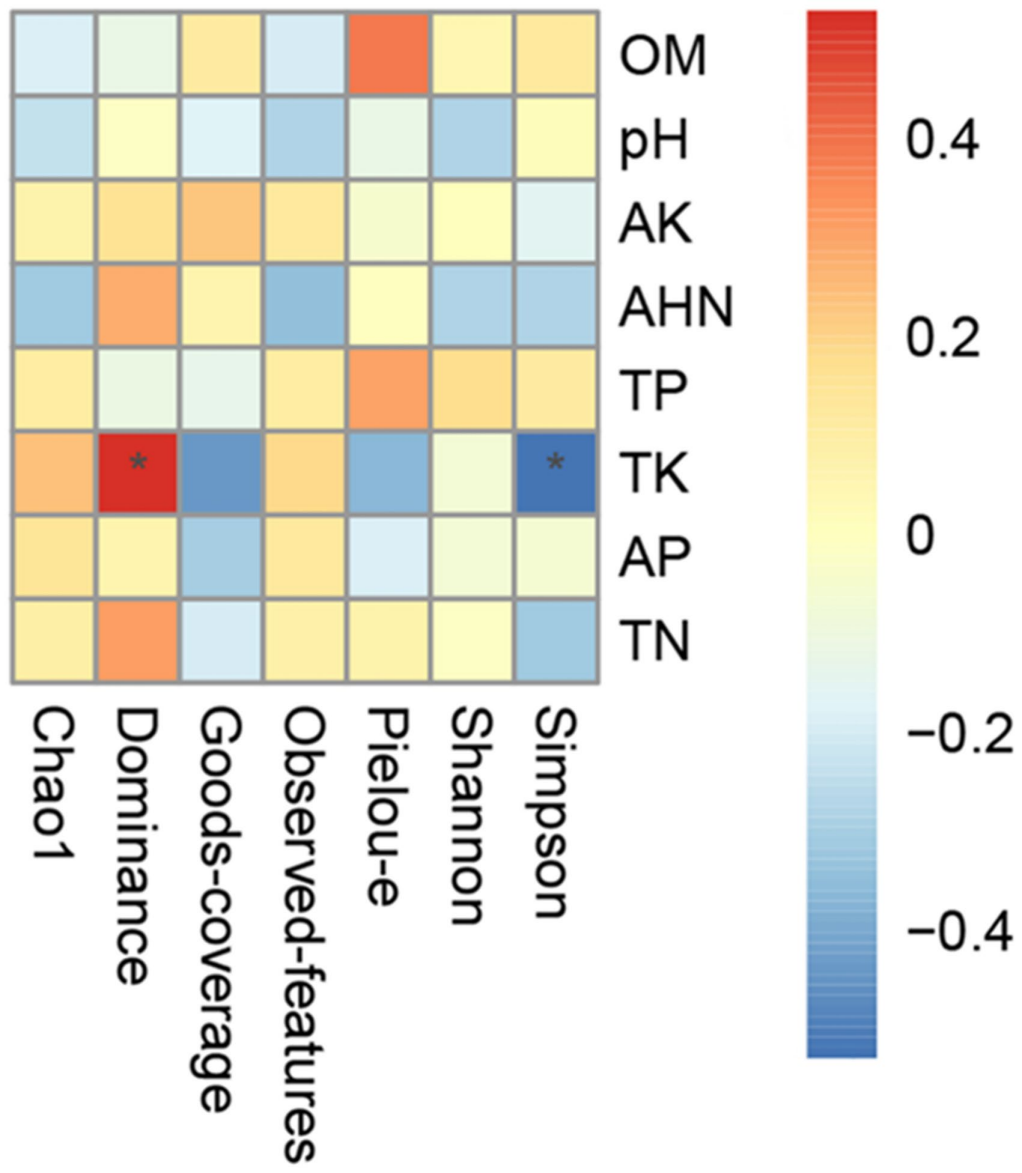
Correlation between soil physicochemical factors and microbial diversity. *indicates significant correlation (*p* < 0.05).

The correlations between soil physicochemical properties and bacterial alpha diversity were characterized using Spearman rank correlation in Perl 5.26.2. The results are visualized as a heatmap, which illustrates the correlation coefficients between the soil properties and the alpha diversity indices (Figure 4). The color gradient (from blue to red) represents negative to positive correlations, with intensity corresponding to the magnitude of the correlation coefficient (ranging from −0.4 to 0.4). Notably, TK showed significant targeted correlations: a negative correlation with the Simpson index (marked by the blue asterisk; *p* < 0.05) and a positive correlation with the Dominance index (marked by the red asterisk; *p* < 0.05). These patterns indicate that higher TK content in rhizosphere soils is linked to increased bacterial community dominance and reduced community diversity. In elevated TK levels, bacterial species effectively utilizing potassium will become dominant in the rhizosphere, whereas potassium-sensitive bacteria can be progressively excluded, thereby decreasing the bacterial diversity. In contrast, other soil properties exhibited weak correlations with all measured alpha diversity indices. These findings suggest that TK is a key edaphic driver shaping the alpha diversity of rhizosphere bacterial communities in this study, exerting a more pronounced impact than the other evaluated soil physicochemical factors.

**Figure 5:**
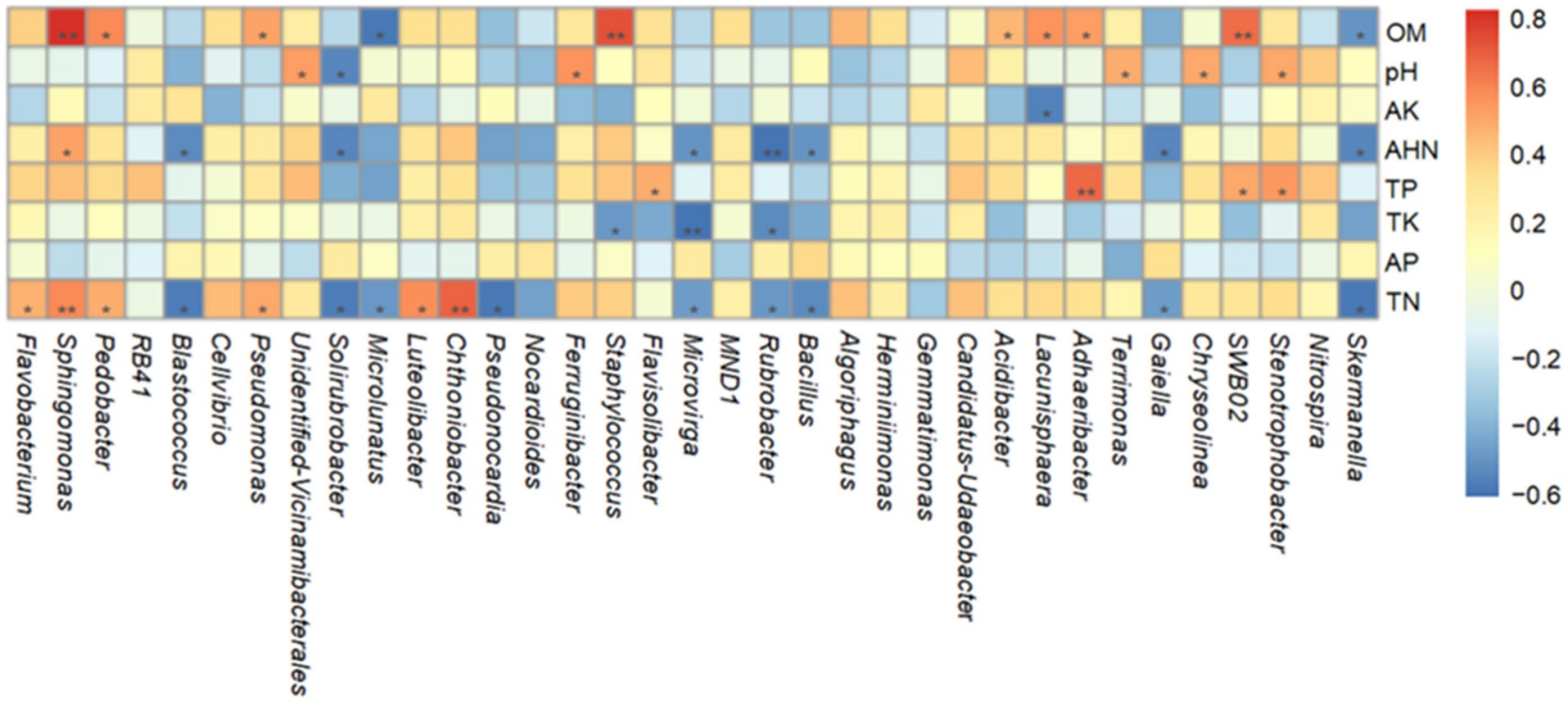
Spearman correlation heatmap of bacteria at the genus level. *Indicates significant correlation (*p* < 0.05); **indicates highly significant correlation (*p* < 0.01).

Spearman correlation analysis was employed to assess the relationships between soil physicochemical properties and dominant bacterial genera in the rhizosphere communities (Figure 5). *Unidentified Vicinamibacterales* showed a significant positive correlation with soil pH (*p* < 0.05). *Sphingomonas* was strongly positively correlated with OM (*p* < 0.01), TN (*p* < 0.01), and AHN (*p* < 0.05). The genera *Pedobacter*, *Pseudomonas*, and *Flavobacterium* were also positively associated with TN. Envfit analysis further indicated that TP (*R*^2^ = 0.76, *p* < 0.01), TN (*R*^2^ = 0.53, *p* < 0.01), and TK (*R*^2^ = 0.52, *p* < 0.01) were strong predictors of bacterial community variation at the genus level. Additionally, AHN moderately influenced community structure (*R*^2^ = 0.31, *p* < 0.05).

## 4 Discussion

The rhizosphere is a critical interface for plant–soil-microbe interactions, and its bacterial communities play key roles in regulating plant growth, nutrient acquisition, and stress resistance (Liu et al., 2022; Liu et al., 2019). Herein, we analyzed the rhizosphere bacterial community structure, diversity, and driving factors of five characteristic Xinjiang medicinal plants. These plants were cultivated continuously in the same plots for 3 years, with shared soil, dense spacing, consistent climate, and standardized management. Such experimental arrangement minimized confounding variables, ensuring microbial variations were driven primarily by plant species rather than environmental or management factors.

### 4.1 Responses of rhizosphere soil properties to the medicinal plant species

The contents of OM, TN, and TP in the rhizosphere of the five medicinal plants were significantly higher than those in non-planted soil (CK). Specifically, root exudates and litter decomposition are key contributors to the accumulation of OM in the plant rhizosphere (Malík and Tlustos, 2025), which explains higher OM content in rhizosphere soils than that in non-planted control. Meanwhile, sugars and amino acids exuded from plant root systems can enhance the bioavailability of soil nitrogen (N) and phosphorus (P) (Smith, 2007; Mazhar et al., 2021), a mechanism that accounts for the elevated TN and TP levels observed in the rhizosphere relative to CK soil. Furthermore, the elevated soil nitrogen content (TN and AHN) can be attributed to special regulation effect of secreted signal molecules from plant roots (Zhu et al., 2024). Notably, TP in rhizosphere soils increased by 2.6–2.9 times (*p* < 0.05), likely due to organic acids in root exudates enhancing phosphorus solubility (Pett-Ridge et al., 2020). Pronounced plant species-specific differences in soil properties were observed: HTR’s OM was significantly higher than other species, and ARL/AEJ had higher TK, while TKR/ARL had higher AK. These results suggest that chemical characteristics of the medicinal plant species actively shape rhizosphere soil properties.

### 4.2 Effect of plant species and soil physicochemical factors on the rhizobacterial community

**Figure 6:**
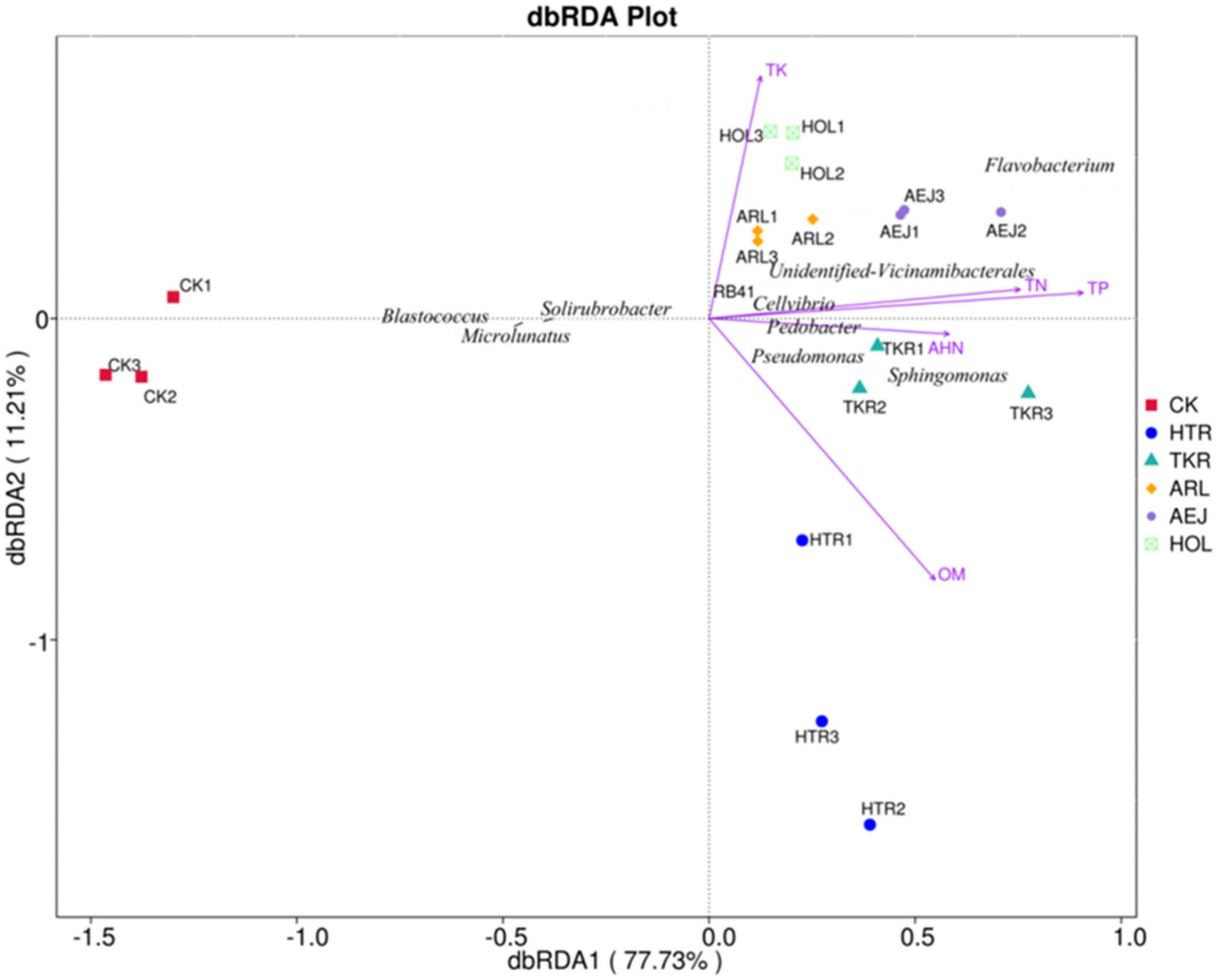
Distance-based redundancy analysis (dbRDA) between the rhizosphere physicochemical properties and bacterial community.

The rhizosphere soils of different medicinal plants exhibited substantial specificity in bacterial diversity and community structure owing to the plant-driven selective bacterial enrichment. The alpha diversity indices of rhizobacterial communities showed significant divergence among five medicinal plants. Venn analysis revealed that all plant rhizospheres contained a remarkably higher proportion of unique ASVs than the shared ASVs. This compelling pattern demonstrated that each plant actively screened and enriched a distinct bacterial combination from the soil microbial community. Subsequent taxonomic analyses further confirmed pronounced compositional and abundance variations of plant-specific rhizosphere bacterial communities. These community differences were closely linked to variations in soil properties. dbRDA correlation analyses (Figure 6) revealed that soil physicochemical properties were key determinants in assembling rhizobacterial communities of medicinal plants. TP, TN, and OM were identified as the three significant factors influencing bacterial community structure. TP exhibited the largest explanatory power in dbRDA analysis, in good agreement with research on *Rheum officinale* continuous cropping systems showing the critical influence of phosphorus level on rhizosphere bacterial community structure and diversity (Wan et al., 2025). Furthermore, microorganisms can acquire and cycle phosphorus through production of phosphatases, organic acid secretion, and expression of high-affinity phosphate transporters (Richardson and Simpson, 2011). Strong correlations between TP and bacterial community likely reflected the rhizosphere bacterial selection by phosphorus availability. TN showed strong positive correlations with multiple beneficial bacterial genera, including *Sphingomonas*, *Pseudomonas*, *Flavobacterium* and *Pedobacter* (Figure 5). Deng et al. (2024) also found that nitrogen availability significantly affected the enrichment of plant growth-promoting rhizobacteria and disease-suppressive taxa in Panax notoginseng systems. Nitrogen not only is an essential nutrient source for microbial growth, but also can regulate root exudation patterns, thereby driving the selective recruitment of specific bacterial communities (Zhu et al., 2024). The enrichment of OM in medicinal plant rhizospheres can create nutrient-rich microhabitats that selectively influence bacterial community assembly (Witzgall et al., 2024). In this work, HTR, with the highest OM content (146.21 g/kg), exhibited distinct bacterial assemblages characterized by the enrichment of *Sphingomonas* (Figures 3, 5). Meanwhile, *Sphingomonas* genus bacteria can facilitate organic compound degradation and plant growth promotion (Asaf et al., 2020). The plant-specific OM enrichment showed differential root exudation and litter decomposition rates among medicinal plants, demonstrating that plant species actively regulate their rhizosphere environments.

The significant negative correlation (*p* < 0.05) between TK and the Simpson index suggested that potassium enrichment may compromise bacterial community diversity. AEJ sample with high TK values exhibited dominant bacterial genus *Sphingomonas*. In contrast, HTR sample with the lowest TK values showed dominant *Flavobacterium* and *Pedobacter* bacterial genera. These results demonstrated that soil TK significantly affected the bacterial composition of these plant species, which aligned with studies on *Moso bamboo* and *Codonopsis pilosula* (Zheng et al., 2022; Huo et al., 2025). The change in composition and structure of rhizosphere bacteria can be partially induced by the competitive exclusion mechanism. The soil conditions with high TK contents will intensify the potassium-related competitive effects among rhizosphere bacteria, thus inhibiting the potassium-sensitive bacteria and reducing the *α*-diversity (Zhongfu et al., 2025). Strategic K-fertilizer management could be a feasible approach to maintain bacterial diversity and enhance soil ecosystem stability.

Additionally, bacterial genus *unidentified Vicinamibacterales* displayed pH-dependent microbial distribution features (Figure 5), which is consistent with the well-established relationship of soil pH and bacterial community structure across multiple medicinal plant systems (Li et al., 2023; Chen et al., 2025). Soil pH can affect membrane transport, enzyme activity, and cellular homeostasis, thus effectively regulating the bacterial community structure (Malik et al., 2017; Puissant et al., 2019). The relatively weak correlation between pH and diversity metrics in this study may be attributed to the narrow pH range among samples (7.30–7.83), which is suitable for the growth of most rhizosphere bacteria (Rousk et al., 2010).

Root exudation patterns can simultaneously shape soil environment and microbial communities. The composition and concentration of root exudates for medicinal plants have inherent differences, enabling distinct nutrient status of each plant rhizosphere and plant-specific enrichment of bacterial taxa (Sasse et al., 2018). Moreover, plants dynamically modulate their associated bacterial communities during different growth stages to meet changing nutritional and protective demands (Reinhold-Hurek et al., 2015). Collectively, these results demonstrate that given the rigorous experimental control of geographical variability, community differentiation can be primarily attributed to plant species through their effects on soil chemistry and root exudate-mediated microbial recruitment.

### 4.3 Ecological functions of rhizosphere bacterial community structure

The rhizosphere soils of all medicinal plants mainly consisted of *Proteobacteria*, *Bacteroidota*, and *Acidobacteriota* at the phylum level, which is well consistent with previous reports in *Paeonia lactiflora* and *Ginseng* (Saleem et al., 2019; Santoyo, 2022). However, the relative abundances of these bacterial phyla exhibited significant variations among different plant species, suggesting distinct bacterial community regulation effects of these medicinal plants, likely mediated by differences in soil nutrient status.

*Proteobacteria*, *Bacteroidota*, and *Acidobacteriota* synergistically participate in soil organic matter decomposition and nutrient cycling, with distinct yet complementary ecological functions. Specifically, *Proteobacteria* has strong environmental adaptability and plays an important role in nitrogen fixation and soil organic matter decomposition (Sharopov et al., 2018); *Bacteroidota* can mediate the decomposition of polysaccharide organic substances in soil by secreting extracellular enzymes (Shi et al., 2023); *Acidobacteriota* contains genes encoding cellulase and hemicellulase, which can degrade lignin and cellulose and metabolize stubborn carbon substrates in soil with limited nutritional resources, thus improving the nutrient content in soil (Tahir et al., 2018; Smit et al., 2001). The *Proteobacteria/Acidobacteriota* ratio (P/A ratio) serves as a partial indicator of nutrient-mediated functional shifts in plant rhizobacterial communities, wherein a high P/A ratio denotes an eutrophic (nutrient-rich) soil condition (Vaghela and Gohel, 2023; Verma et al., 2022). Compared to ARL and HOL, HTR, TKR and AEJ exhibited significantly increased P/A ratios owing to their higher organic matter content, suggesting that nutrient-rich rhizosphere environments favored the formation of copiotrophic bacterial communities. On the other hand, the enrichment of *Acidobacteriota* in ARL and HOL may be attributed to organic acid secretion of plants for adapting nutrient-limited conditions. Therefore, medicinal plants can obtain functionally specific bacterial assemblages via the control of soil nutrients.

At the family and genus levels, *Chitinophagaceae* were ubiquitously presented in five medicinal plant rhizospheres, and *Flavobacteriaceae* exhibited higher relative abundance in TKR and AEJ. Carrión et al. (2019) demonstrated that bacteria of *Chitinophagaceae* and *Flavobacteriaceae* family became enriched during pathogenic fungal invasion owing to their unique capabilities in enhancing enzymatic activities related to fungal cell wall degradation and secondary metabolite biosynthesis encoded by non-ribosomal peptide synthetases (NRPS) and polyketide synthases (PKS). Moreover, *Chitinophagaceae* showed significant positive correlations with β-glucosidase activity (Xiong et al., 2009), crucially affecting the organic matter decomposition. The genus *Sphingomonas* was particularly enriched in ARL and HTR rhizospheres. This bacterial genus can degrade both natural and xenobiotic compounds and promote plant growth via producing the phytohormones, siderophores, and metal chelators (Yang et al., 2009; Yuan et al., 2022). The genus *Pseudomonas* exhibited relatively high abundance in TKR rhizosphere, facilitating the improvement of plant disease resistance mechanisms (Liu et al., 2024). The rhizospheres of ARL and HOL were notably rich in *RB41* taxa. Previous report confirmed that *RB41* can maintain soil metabolic and biogeochemical functions (Zhang et al., 2016), thereby enhancing nutrient utilization efficiency of plants under nutrient-limited conditions. The *Sphingobacterium* and *Cellvibrio* genera exhibited high cellulolytic activity, which were enriched in TKR and AEJ rhizosphere, respectively. Consequently, these results confirmed the specific enrichment of functional bacterial communities closely associated with growth and health of medicinal plants, contributing to enhancing the environmental adaptability of medicinal plants.

Preliminary PICRUSt2-based functional prediction provided additional insights into the metabolic potential of these rhizosphere communities (Supplementary Figures 2, 3). Despite the highest organic matter content in HTR, the genes associated with carbon degradation pathways (e.g., starch and sucrose metabolism, amino sugar metabolism) were enriched in TKR and AEJ rather than in HTR, suggesting that organic matter alone is insufficient to drive decomposition functions without concurrent nutrient availability (López-Mondéjar et al., 2020). Similarly, classical stress response genes (Kdp system, ProVWX, BADH) were most abundant in nutrient-poor CK rather than in high-TK ARL and AEJ. Interestingly, antioxidant pathways genes were rich in ARL and AEJ samples, such as the pentose phosphate pathway and ascorbate metabolism (Stincone et al., 2015). These results imply that potassium-rich environment may induce oxidative stress, which may be attributed to alternative adaptation of rhizosphere bacteria. These findings demonstrate that microbial functional responses are shaped by complex interactions among multiple soil properties rather than single factors in isolation (Quintero-Yanes et al., 2024).

## 5 Conclusion

This study systematically analyzes the rhizosphere soil physicochemical properties and bacterial community structure of five medicinal plants native to Xinjiang (HTR, TKR, ARL, AEJ and HOL). Compared with non-planted soil (CK), the rhizosphere samples of medicinal plants exhibit elevated organic matter (OM), total nitrogen (TN), and total phosphorus (TP) contents, suggesting the active regulatory effects of plant growth on soil physicochemical properties. Furthermore, the high-throughput sequencing results exhibit unique ASVs of 34.04–46.70% for plant rhizosphere and CK soils, revealing the enrichment of plant-specific microorganisms in each medicinal plant. Specifically, *Proteobacteria*, *Bacteroidota* and *Acidobacteriota* are core bacterial assemblages in the rhizosphere at the phylum level, while *Actinobacteriota* dominates in CK. Moreover, the genera *Chitinophagaceae*, *Sphingomonas*, *Pseudomonas*, and *RB41* are selectively enriched in different medicinal plant rhizospheres. Correlation analysis between soil physicochemical properties and bacterial alpha diversity identifies that TN/TP are critical driving factors shaping dominant genera of bacterial communities, while TK may decline the rhizosphere bacterial diversity of studied medicine plants. These results are instructive for the optimized fertilization and the development of targeted microbial inoculant in the medicinal plant cultivation.

## Data Availability

The datasets presented in this study can be found in online repositories. The names of the repository/repositories and accession number(s) can be found in the article/Supplementary material.

## Supplementary material

SUPPLEMENTARY FIGURE 1 Rarefaction curves based on the 16S rRNA gene sequencing.

SUPPLEMENTARY FIGURE 2 Bar plot showing the relative abundance of genes in carbohydrate metabolism-related pathways.

SUPPLEMENTARY FIGURE 3 Bar plot of relative abundance of genes responsive to high potassium stress.
