# Supplementary figures and images for: Comparative analysis of rhizobacterial communities across five medicinal plants in Xinjiang
Source: Front Microbiol. 2026 Apr 22;17:1785383. doi: 10.3389/fmicb.2026.1785383 (PMC13144048; doi:10.3389/fmicb.2026.1785383)

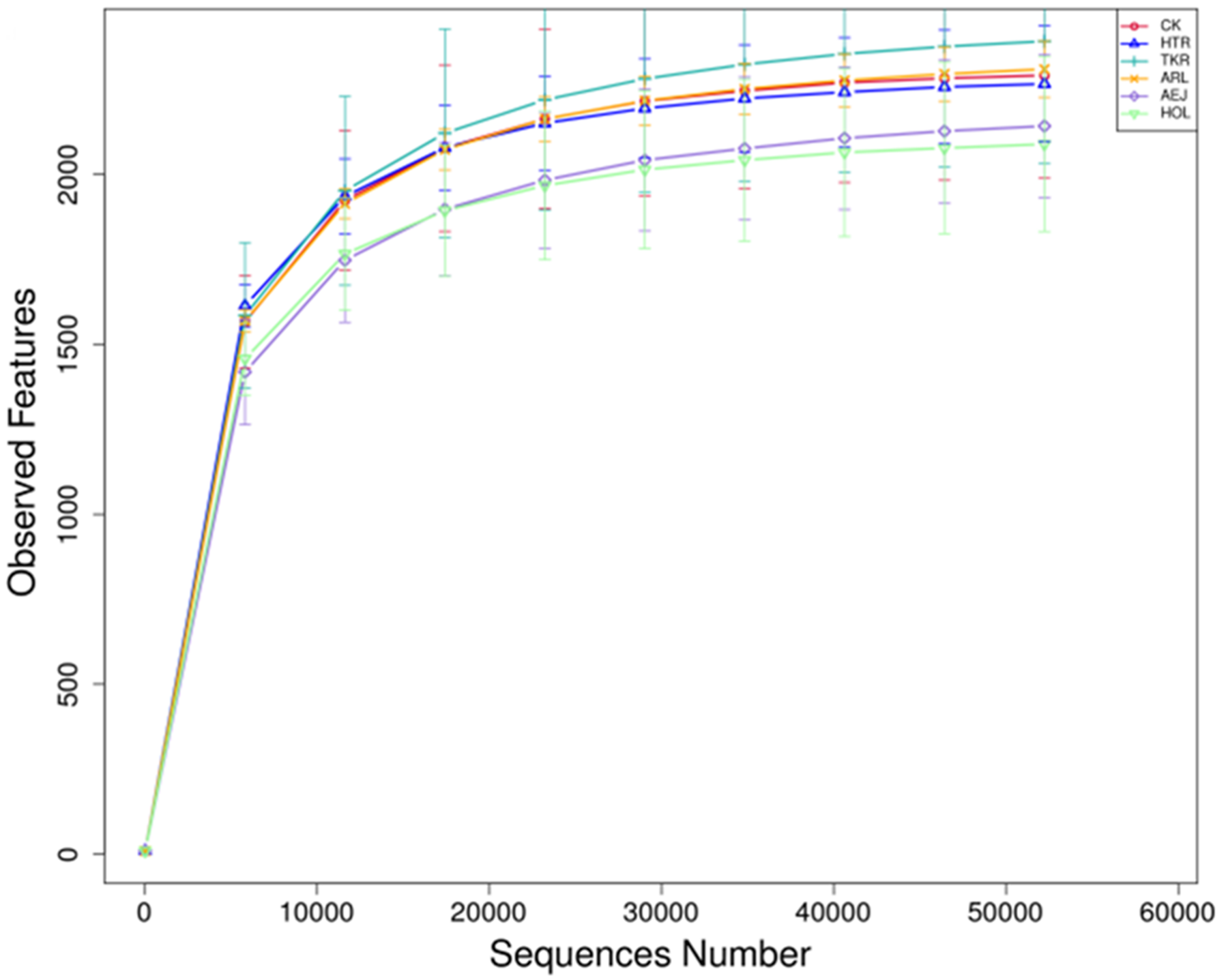

Supplement: SUPPLEMENTARY FIGURE 1 — Rarefaction curves based on the 16S rRNA gene sequencing. [file Image_1.tif]

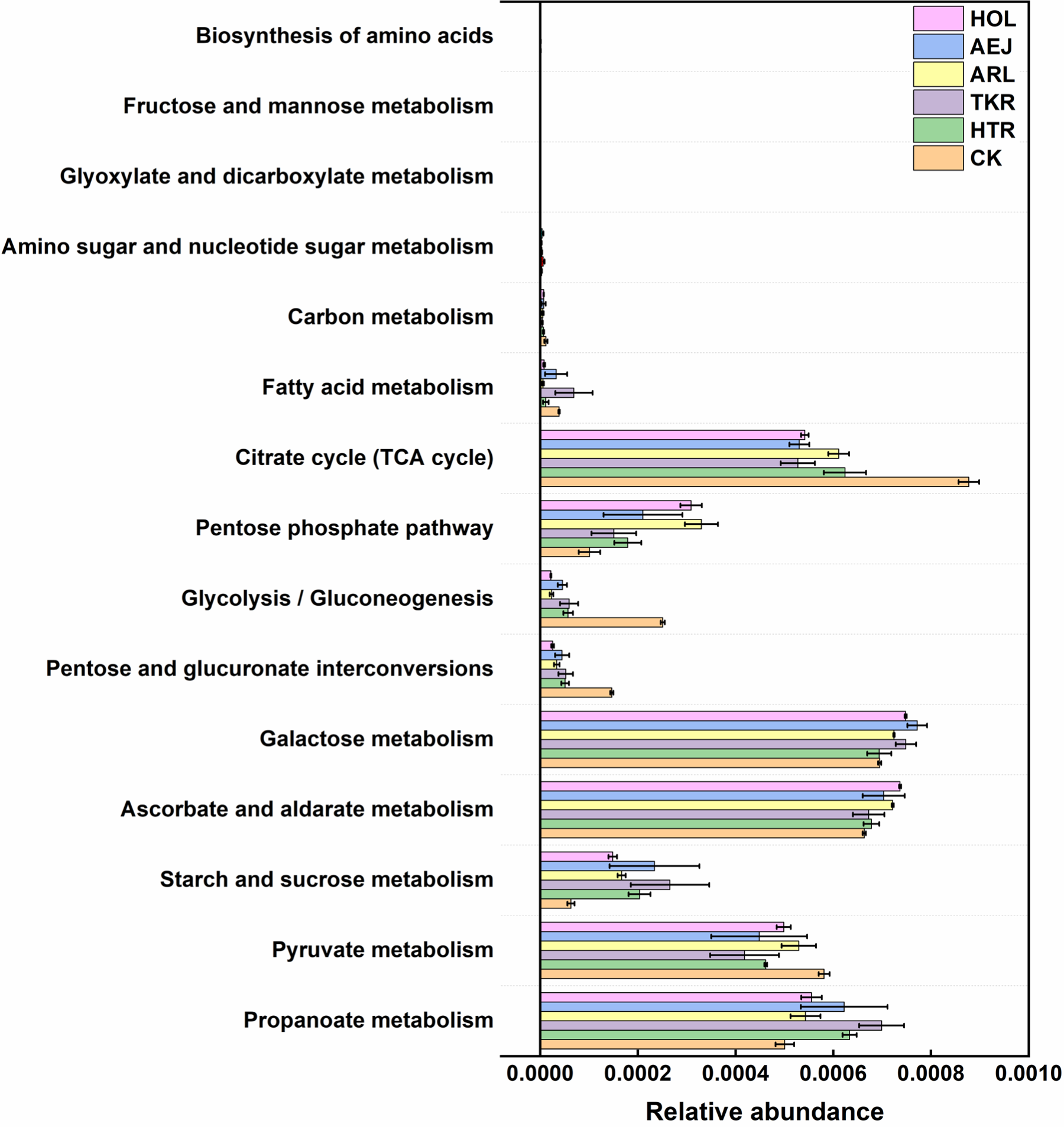

Supplement: SUPPLEMENTARY FIGURE 2 — Bar plot showing the relative abundance of genes in carbohydrate metabolism-related pathways. [file Image_2.tif]

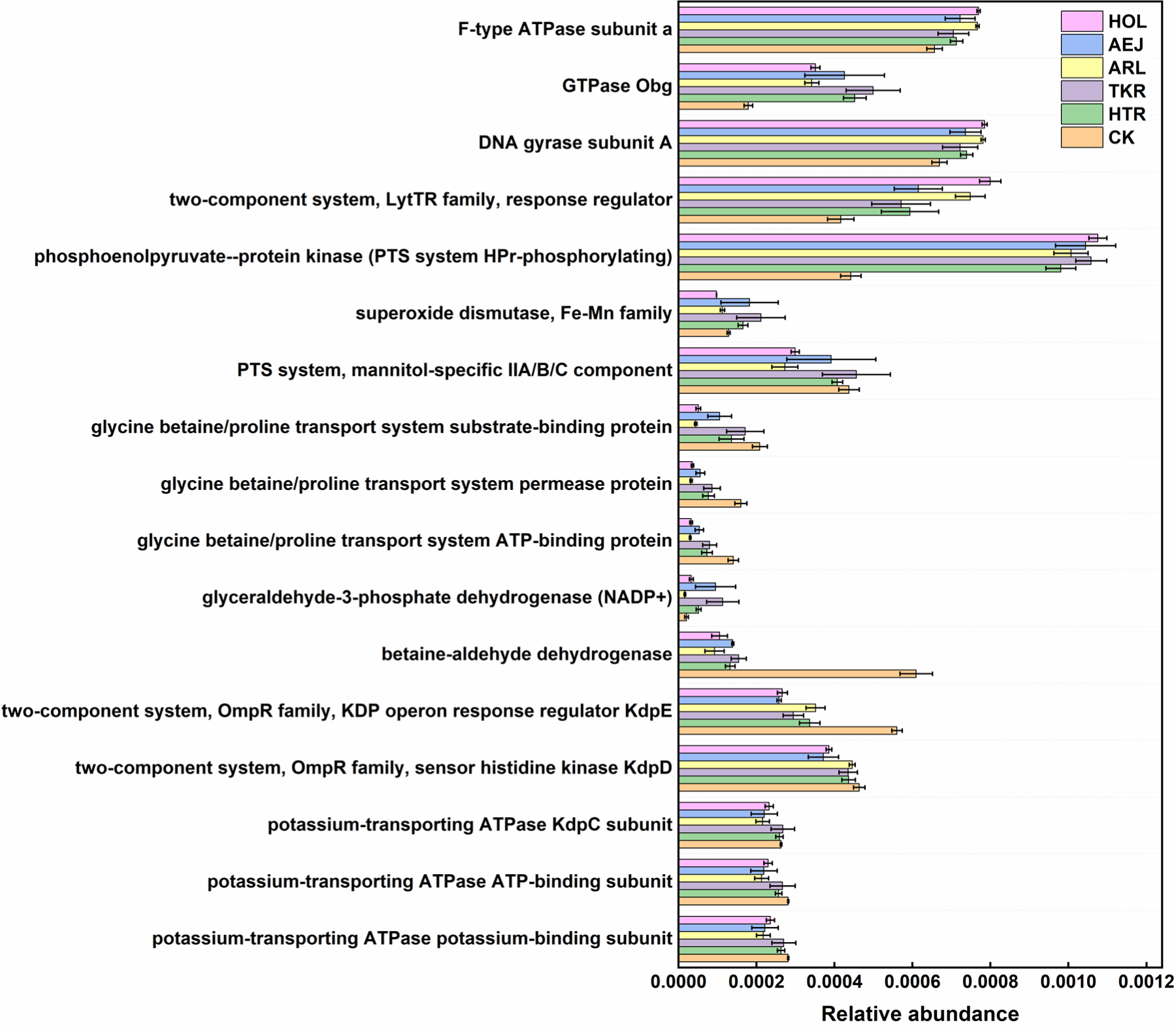

Supplement: SUPPLEMENTARY FIGURE 3 — Bar plot of relative abundance of genes responsive to high potassium stress. [file Image_3.tif]
