# Supplementary material for: Comparative analysis of rhizobacterial communities across five medicinal plants in Xinjiang
Source: Front Microbiol. 2026 Apr 22;17:1785383. doi: 10.3389/fmicb.2026.1785383 (PMC13144048; doi:10.3389/fmicb.2026.1785383)
Supplement: Supplementary file 4 [file Table_1.docx]

**Supplementary Table 1.** Statistics of sequencing data of five soil samples.

| Sampled Rhizosphere | Raw tags | Effective tags | Effective (%) | ASVs | Goods-coverage |
| --- | --- | --- | --- | --- | --- |
| CK | 103957±1947 | 79989±7627 | 76.98±7.92 | 2291±371 | 0.9983±0.0005 |
| HTR | 104432±19989 | 76249±8024 | 72.97±1.37 | 2254±211 | 0.9983±0.0012 |
| TKR | 131460±8319 | 100330±7982 | 76.28±2.35 | 2378±441 | 0.9980±0.0010 |
| ARL | 123869±8171 | 98085±4214 | 79.47±7.15 | 2294±99 | 0.9977±0.0006 |
| AEJ | 132440±992 | 104944±5126 | 79.25±4.26 | 2135±258 | 0.9983±0.0006 |
| HOL | 114721±29108 | 88824±9328 | 77.28±0.87 | 2082±314 | 0.9979±0.0008 |
