# Supplementary material for: Comparative analysis of rhizobacterial communities across five medicinal plants in Xinjiang
Source: Front Microbiol. 2026 Apr 22;17:1785383. doi: 10.3389/fmicb.2026.1785383 (PMC13144048; doi:10.3389/fmicb.2026.1785383)
Supplement: Supplementary file 5 [file Table_2.docx]

**Supplementary Table 2.** Diversity of bacterial communities in five soil samples.

| Sampled Rhizosphere | Chao1 | Pielou-e | Shannon | Simpson |
| --- | --- | --- | --- | --- |
| CK | 2305±378^a^ | 0.902±0.007^ab^ | 10.052±0.210^a^ | 0.998±0.001^ab^ |
| HTR | 2288±245^a^ | 0.924±0.005^a^ | 10.291±0.126^a^ | 0.999±0.001^a^ |
| TKR | 2433±479^a^ | 0.898±0.259^ab^ | 10.058±0.543^a^ | 0.997±0.001^ab^ |
| ARL | 2347±108^a^ | 0.910±0.020^ab^ | 10.157±0.071^a^ | 0.998±0.001^ab^ |
| AEJ | 2192±283^a^ | 0.877±0.034^b^ | 9.700±0.531^a^ | 0.996±0.003^b^ |
| HOL | 2127±312^a^ | 0.910±0.004^ab^ | 10.026±0.170^a^ | 0.998±0.001^ab^ |

Distinct letters (a, b) denote significant differences of parameters (P < 0.05).
